# Enhancing stereotactic ablative boost radiotherapy dose prediction for bulky lung cancer: A multi‐scale dilated network approach with scale‐balanced structure loss

**DOI:** 10.1002/acm2.14546

**Published:** 2024-10-07

**Authors:** Lei Huang, Xianshu Gao, Yue Li, Feng Lyu, Yan Gao, Yun Bai, Mingwei Ma, Siwei Liu, Jiayan Chen, Xueying Ren, Shiyu Shang, Xuanfeng Ding

**Affiliations:** ^1^ Department of Radiation Oncology Peking University First Hospital Beijing China; ^2^ Department of Medical Biochemistry and Biophysics Karolinska Institutet Stockholm Sweden; ^3^ National Cancer Centre/National Clinical Research Centre for Cancer/Cancer Hospital Chinese Academy of Medical Sciences and Peking Union Medical College Beijing China; ^4^ Department of Radiation Oncology William Beaumont University Hospital, Cordell Health Royal Oak Michigan USA

**Keywords:** bulky lung cancer, deep learning, partial stereotactic ablative boost radiotherapy, three‐dimensional dose prediction

## Abstract

**Purpose:**

Partial stereotactic ablative boost radiotherapy (P‐SABR) effectively treats bulky lung cancer; however, the planning process for P‐SABR requires repeated dose calculations. To improve planning efficiency, we proposed a novel deep learning method that utilizes limited data to accurately predict the three‐dimensional (3D) dose distribution of the P‐SABR plan for bulky lung cancer.

**Methods:**

We utilized data on 74 patients diagnosed with bulky lung cancer who received P‐SABR treatment. The patient dataset was randomly divided into a training set (51 plans) with augmentation, validation set (7 plans), and testing set (16 plans). We devised a 3D multi‐scale dilated network (MD‐Net) and integrated a scale‐balanced structure loss into the loss function. A comparative analysis with a classical network and other advanced networks with multi‐scale analysis capabilities and other loss functions was conducted based on the dose distributions in terms of the axial view, average dose scores (ADSs), and average absolute differences of dosimetric indices (AADDIs). Finally, we analyzed the predicted dosimetric indices against the ground‐truth values and compared the predicted dose‐volume histogram (DVH) with the ground‐truth DVH.

**Results:**

Our proposed dose prediction method for P‐SABR plans for bulky lung cancer demonstrated strong performance, exhibiting a significant improvement in predicting multiple indicators of regions of interest (ROIs), particularly the gross target volume (GTV). Our network demonstrated increased accuracy in most dosimetric indices and dose scores in different ROIs. The proposed loss function significantly enhanced the predictive performance of the dosimetric indices. The predicted dosimetric indices and DVHs were equivalent to the ground‐truth values.

**Conclusion:**

Our study presents an effective model based on limited datasets, and it exhibits high accuracy in the dose prediction of P‐SABR plans for bulky lung cancer. This method has potential as an automated tool for P‐SABR planning and can help optimize treatments and improve planning efficiency.

## 1 INTRODUCTION

Bulky lung cancer, which is defined as lung cancer cases involving lesions with a diameter of 4.5 cm on computed tomography (CT) scans, poses significant challenges owing to the high tumor burdens and close proximity to surrounding normal organs.^1^ Conventional fractionated radiotherapy (CFRT) offers limited efficacy, resulting in low long‐term patient survival rates.^2^, ^3^ While stereotactic ablative boost radiotherapy (SABR) is promising for improving local tumor control, its standalone application is still restricted, especially for advanced non‐small cell lung cancer, owing to significantly increased radiation toxicity and adverse reactions.^4^, ^5^, ^6^ Thus, effective local control remains a critical clinical challenge for bulky lung cancer.

To improve this outcome, partial stereotactic ablative boost radiotherapy (P‐SABR) has been proposed as a joint treatment strategy involving CFRT and SABR.^1^ P‐SABR comprises a two‐stage plan: the application of localized SABR to deliver a high dose within the tumor and the application of CFRT to supplement the prescribed dose for the entire gross target volume (GTV).^1^ A retrospective clinical study demonstrated that P‐SABR significantly increased the bioequivalent dose (BED) to the tumor center (reaching 107.3 Gy) and achieved a local control rate of 85.7% after 2 years for bulky non‐small cell lung cancer without increasing the incidence of severe acute side effects.^1^ Although P‐SABR has exhibited highly effective therapeutic outcomes, planning is time‐consuming, as it involves preliminary determination of the gross tumor boost volume (GTVb) by radiation oncologists, plan design and dose distribution calculations by physicists, and iterative modification of the GTVb by radiation oncologists to optimize tumor coverage while minimizing harm to the surrounding tissues. Repetitive dose distribution calculations prolong the planning time, therefore limiting the clinical utility of P‐SABR. Hence, in practice, developing an advanced method that enables radiotherapists to rapidly evaluate whether the dose distribution within the present GTVb conforms to clinical requirements throughout the iterative procedure is pivotal for the implementation of P‐SABR technology.

With the rapid development of artificial intelligence, sophisticated deep learning networks, such as residual networks,^7^, ^8^ U‐Net, and their variants,^9^, ^10^, ^11^, ^12^, ^13^, ^14^, ^15^, ^16^, ^17^, ^18^, ^19^ generative adversarial networks (GANs),^20^, ^21^, ^22^ and transformers,^23^, ^24^ have been applied to dose prediction. The predicted dose distributions can be utilized to determine optimal and applicable treatments.^13^ These networks have demonstrated outstanding predictive performance across various cancer types, such as head and neck, prostate, and cervical cancers. However, applying these networks to P‐SABR plans for bulky lung cancers poses unique challenges. Interpatient variability in tumor size necessitates the network's ability to capture multi‐scale features. In addition, varying tumor locations among bulky lung cancer patients result in significant differences in the distances between the tumor and organs‐at‐risk (OARs). These features result in data with many multi‐scale characteristics, which poses difficulties for the application of convolutional neural networks (CNNs).^25^

In this study, we identified the rich multi‐scale features of P‐SABR plans for bulky lung cancer by analyzing their data characteristics. Given the current lack of networks designed specifically for extracting and utilizing multi‐scale features, dose prediction performance can be potentially improved by enhancing the multi‐scale feature extraction and utilization capabilities of existing advanced networks. Recent studies have also explored multi‐task training modes to improve the effectiveness of dose distribution prediction networks, using overall MSE‐Loss as the main task, MSE‐Loss of other regions of interest (ROIs), and dosimetric indices as auxiliary tasks.^19^, ^26^, ^27^ Many of these studies employ fixed weight training, but advancements in deep learning have introduced methods to improve multi‐task learning performance.^28^, ^29^, ^30^, ^31^ Based on these considerations, we propose a novel method to enhance the accuracy of dose distribution prediction for P‐SABR plans for bulky lung cancers. First, we introduce a multi‐scale dilated network (MD‐Net) to capture and leverage multi‐scale features in bulky lung cancer patient datasets. Second, we optimize the loss function by not only introducing but also balancing the impacts of various ROIs in the dose‐prediction task. Our results demonstrate the effectiveness of this deep learning approach in predicting doses for P‐SABR plans in bulky lung cancer cases, suggesting its potential to streamline P‐SABR and even automate the generation of the GTVb.

## 2 MATERIALS AND METHODS

### 2.1 Patient data

For this study, a total of 74 sets of data on patients with bulky lung cancer treated with P‐SABR in Department of Radiation Oncology, Peking University First Hospital were collected. The data for each patient included three‐dimensional (3D) CT images, contoured structures, and 3D dose distributions. The contoured structures for each patient encompassed at least eight extractable structures: body, heart, lungs, esophagus, trachea, spinal cord, planning gross target volume (PGTV), and GTVb. The GTVb for each patient was determined through repeated iteration and optimization. The specific process is outlined as follows. First, the radiation oncologist preliminarily defines the GTVb, and then the physicist develops the treatment plan and performs the dose calculations. The radiation oncologist then reviews the dose calculation results. If the dose to the nearby OARs exceeds the dose limit, the radiation oncologist should reduce the volume of the GTVb. Conversely, if the dose to the OARs is within the limits, the radiation oncologist may expand the GTVb. This process aims to delineate the largest possible GTVb, thereby increasing the dose to the GTV. The P‐SABR plans were volumetric modulated arc therapy (VMAT) plans generated via the Eclipse treatment planning system (V13.5, Varian Medical System, Palo Alto, California, USA), aiming at delivering 32 Gy to the GTVb in four fractions.

### 2.2 Data preparation

Data preprocessing and augmentation were conducted using neural networks to ensure accurate dose distribution predictions.

During preprocessing, the contours of the structures were extracted and converted into 3D binary masks. Each voxel contained within the contour was assigned a value of 1; the remaining voxels were all assigned a value of 0. To address the different spatial resolutions and ranges among the CT data, structure masks, and dose distributions for each patient, all data were resampled into a 3D tensor of size 128 × 128 × 64 using zero padding with voxel dimensions of 5 × 5 × 2.5 mm. When resampling, sampling positions along the patient's vertical axis were selected to position the layers containing the PGTV and GTVb at the center of the tensor. This operation aligned the spatial distributions of different patient datasets, enabling the neural network to focus on learning the underlying dose distribution pattern and mitigating the effect of irrelevant spatial changes. The top 0.5% of the CT data with the highest value for each patient were truncated to remove lead points and other noise with high CT values, reduce the interference of outliers on the network and the difficulty of training, and improve the robustness of the network. All truncated CT and dose distribution data were approximately normalized to the [0,1] range. Normalization eliminated data distribution deviations among all types of data and accelerated network convergence. Overall, the final constructed input was a tensor with dimensions of 9 × 128 × 128 × 64, comprising nine channels: body, heart, lungs, esophagus, trachea, spinal cord, PGTV, GTVb, and CT data. The dose distribution was reshaped into a tensor with dimensions of 1 × 128 × 128 × 64 as the ground‐truth.

For augmentation, random translation ([‐50 mm, +50 mm]) and rotation ([‐10°, +10°]) were applied to accommodate varying tumor distributions and maintain body flatness. A random flipping data augmentation method was introduced to address the data imbalance between patients with left and right lung cancers. Specifically, 60% of the training data underwent rotation, 60% underwent translation, and 20% underwent flipping operations, significantly enlarging the original training dataset for an improved training process.

### 2.3 Network architecture

The data characteristics of bulky lung cancer patients are different from those of patients with other types of tumors, primarily because of the variations in tumor sizes and the relative positions of the tumors and OARs among patients. The details are provided in Table 1. The differences in the sizes of tumors and their relative distances profoundly impact the dose distributions.

**TABLE 1:** Statistics of tumor sizes and distances to the centers of various serial OARs.

	Min	Median	Max	Mean ± Std
Size of PGTV (cm^3^)	41.0578	164.6958	681.3676	213.6178 ± 157.0096
Size of GTVb (cm^3^)	1.0081	18.8679	314.4993	35.5896 ± 48.8985
Distance from PGTV to esophagus (cm)	1.7524	6.4331	11.6827	6.3842 ± 1.9994
Distance from PGTV to trachea (cm)	2.4193	6.6395	11.3615	6.8023 ± 2.2120
Distance from PGTV to spinal cord (cm)	3.7729	7.3204	13.5693	7.6931 ± 2.1165
Distance from GTVb to esophagus (cm)	1.6933	6.6461	12.2306	6.6886 ± 1.9552
Distance from GTVb to trachea (cm)	2.8400	6.9394	11.1417	7.1025 ± 2.1077
Distance from GTVb to spinal cord (cm)	4.2964	7.5360	13.9572	7.9070 ± 2.1676

CNNs face challenges in handling scale changes,^25^ as the shape and spatial distance alterations of objects pose difficulties for capturing features. Previous studies have shown that multi‐scale input networks,^32^, ^33^ multi‐scale feature extraction and fusion networks,^34^, ^35^, ^36^ and multi‐scale output networks^37^ are effective in introducing and using multi‐scale information. Hence, their utilization improves the performance of networks when processing data on complex scales.

**FIGURE 1:**
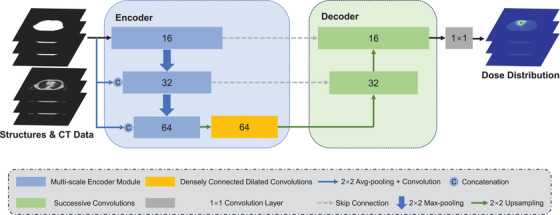
Structure of MD‐Net for the dose prediction of P‐SABR plans for bulky lung cancer. Compared with the structure of U‐Net, which has similar styles, MD‐Net has fewer sampling steps, similar to DD U‐Net. Additionally, the network has three important components: multi‐scale input modules, multi‐scale encoding modules, and densely connected dilated convolutions, which are used to extract and use multi‐scale features.

We propose a multi‐scale dilated network (MD‐Net) that incorporates a multi‐scale input module and multi‐scale feature extraction and processing module (shown in Figure 1) to comprehensively determine the characteristics of tumors of different sizes and the mutual influence between the GTV and OARs at different distances in P‐SABR data. MD‐Net employs an encoding‐decoding architecture comprising three key components: multi‐scale input modules, multi‐scale encoding modules, and densely connected dilated convolutions.

In the encoder path, three multi‐scale encoder modules (with channel sizes of 16, 32, and 64) extract and fuse multi‐scale features from the input CT images and structure masks. These modules accept inputs from the previous multi‐scale encoder module and network at different scales. The output from the last encoder module is passed to the densely connected dilated convolution module for further multi‐scale feature utilization. Skip connections link the encoder and decoder paths.

**FIGURE 2:**
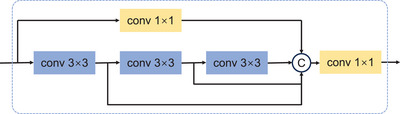
Overview of the multi‐scale encoder module. The input feature map can extract features of different scales (3, 5, and 7) through different paths. The features of each scale are concatenated and fused before continuing to be transmitted to other parts of the network.

**FIGURE 3:**
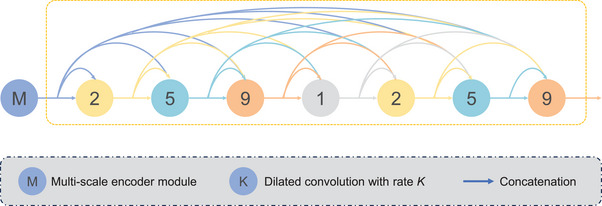
Overview of the densely connected dilated convolutions. The feature maps of the same size output from the multi‐scale encoding module are used as inputs for densely connected convolutions. Convolutional layers with different receptive fields can help capture more complex feature representations.

In the decoder path, two upsampling layers, followed by consecutive convolutional layers, recover the feature maps to the network resolution. The kernel size of all convolutional layers is 3 × 3. This is followed by batch normalization (BN) and rectified linear unit (ReLU) activation. The final 1 × 1 convolutional layer predicts the dose distribution, which is truncated to a reasonable range. More details on the MD‐Net modules are provided in the following text and Figures 2 and 3.

#### 2.3.1 Multi‐scale encoder module

Inspired by the MultiRes block,^36^ we propose a multi‐scale encoder module to extract multi‐scale features from the input feature maps (Figure 2). The input feature map is fed into three consecutive convolutional layers with 3 × 3 kernels and one convolutional layer with a 1 × 1 kernel. The output is concatenated, and further feature fusion is performed using a convolutional layer with a 1 × 1 kernel. These three layers enable the learning and integration of features at scales of 3, 5, and 7 to facilitate a comprehensive understanding of tumor size and its relationship with OARs in dose prediction.

#### 2.3.2 Multi‐scale input module

Inspired by M‐Net,^33^ we employ a multi‐scale input module to construct a data pyramid for the fusion of receptive fields at different scales. The average pooling layer downsamples the CT data and structure masks. The generated low‐resolution images are then fed into a convolutional layer with 3 × 3 kernels. The output is concatenated with feature maps with the same resolution obtained from the multi‐scale encoder module and max pooling layer in the encoder.

#### 2.3.3 Densely connected dilated convolutions

Densely connected dilated convolutions have achieved excellent dose prediction results and can effectively increase the receptive field of the model without increasing the computational complexity or introducing downsampling. Inspired by previous studies, such as DD U‐Net,^19^, ^26^, ^38^, ^39^ we modified the input of the dilated convolutions with a multi‐scale encoder module coupled with various dilation rates (1, 2, 5, and 9) and wider channels to avoid grid effects and extract more information with different scales. Large receptive field convolutions fuse features and analyze global information. Figure 3 illustrates the specific structural design.

### 2.4 Networks for comparison

Various networks capable of focusing on information at different scales were compared to the MD‐Net.

**U‐Net**: A classic network widely used for dose prediction.^9^, ^10^, ^11^, ^12^

**At. Res U‐Net**: Oktay et al. introduced the Attention U‐Net,^40^ which emphasizes features of various sizes. Osman et al. further enhanced this concept using the Attention Res U‐Net (At. Res U‐Net) in the field of dose prediction,^18^ which exhibits superior performance in target dose prediction compared to the Attention U‐Net.

**DD U‐Net**: This network utilizes densely connected dilated convolutions to assist in acquiring features across different scales.^19^, ^26^, ^38^, ^39^, ^41^

### 2.5 Loss function

When using neural networks for dose prediction, the mean squared error loss (MSE‐Loss) is used as the loss function,^11^, ^12^, ^13^ and its formula is

(1) $$\begin{array}{c} \begin{array}{ccc} & & Loss\left(\mathrm{Dos}e_{predict},\;\mathrm{Dos}e_{gt}\right)\\ & & \;\;\;\;=\frac{1}{n}\;\sum_{i=1}^{n}{\left({\mathrm{dose}}_{predict}^{i}-{\mathrm{dose}}_{gt}^{i}\right)}^{2}, \end{array} \end{array}$$

where $\mathrm{dos}e_{gt}$ is the ground‐truth dose value of the voxel, $\mathrm{dos}e_{predict}$ is the predicted dose value of the voxel, *i* is the voxel index, and *n* is the total number of voxels located within the patient's body.

However, voxel importance varies, as accurately predicting voxels within the GTV or OARs is more valuable. A common approach to address this issue involves adding a weighted MSE‐Loss for each ROI atop the overall MSE‐Loss.^19^, ^27^ However, with numerous ROIs, each of which possesses distinct MSE‐Loss values, simple weighting becomes challenging, hinders appropriate weight determination, and potentially disrupts the training process for different ROIs and even overall training tasks. To overcome this, inspired by MetaBalance,^28^ we refined the loss function to align the MSE‐Loss of each ROI with the scale before the weighted summation. The formulas are:

(2) $$\begin{array}{ccc} & & Loss_{ROI}\left(\mathrm{Dos}e_{predict},\;\mathrm{Dos}e_{gt}\right)\\ & & =\frac{1}{n}\;\sum_{i=1}^{n}{\left(dose_{predict,\;ROI}^{i}-dose_{gt,ROI}^{i}\right)}^{2}, \end{array}$$

(3) $$\begin{array}{ccc} & & Loss\left(\mathrm{Dos}e_{predict},\;\mathrm{Dos}e_{gt}\right)=Loss_{Body}\\ & & +\sum_{j=1}^{k}weight_{j}*\frac{Loss_{j}*\left|Loss_{Body}\right|}{\left|Loss_{j}\right|}, \end{array}$$

where *j* is the index of the ROI, *k* is the total number of ROIs $,\;weight_{j}$ is the weight of the *j*th ROI, the weight of the OARs is set to 0.1, and the weight of the targets that is difficult to train is set to 0.4.

### 2.6 Training and validating the model

The patient dataset was randomly divided into a training set (51 plans), a validation set (7 plans), and a testing set (16 plans). All models were trained on two 3090 GPUs with 24 GB of memory, using Adam as the optimizer with default parameters. The initial learning rate was set to 2e‐4, and the cosine annealing strategy was utilized to adjust the learning rate. The weight decay was set to 5e‐5. The batch size was set to four, and the models were trained for over 180 epochs.

### 2.7 Evaluation

Different networks were compared regarding performance on the testing dataset comprising 16 patients. The main indicators for comparison included the following: (1) the average dose score (ADS, the mean absolute error between the ground‐truth and predicted dose values) for each ROI, which is defined as

(4) $$\begin{array}{c} \begin{array}{ccc} & & Average\;Dose\;Score\;\left(ROI\right)\\ & & =\frac{1}{k}\;\sum_{j=1}^{k}\frac{1}{n}\sum_{i=1}^{n}\left(\left|dose_{predict,\;ROI}^{i}-dose_{gt,ROI}^{i}\right|\right), \end{array} \end{array}$$

where *j* is the index of the patient, and *k* is the total number of patients, and (2) the average absolute difference of the dosimetric indices (AADDIs), which is expressed as

(5) $$\begin{array}{c} \begin{array}{ccc} & & Average\;Absolute\;Difference\;of\;Dosimetric\;Indice\\ & & =\frac{1}{k}\;\sum_{j=1}^{k}\left(\left|DI_{predict}-DI_{gt}\right|\right), \end{array} \end{array}$$

where $DI_{predict}$ and $DI_{gt}$ are the predicted and ground‐truth values of the various dosimetric indices for different ROIs, respectively.

Furthermore, the predicted results of the optimal model and the ground‐truth plan were compared across various dosimetric indices. Statistical testing methods (either the paired *t*‐test or the Wilcoxon signed‐rank test, depending on the normality assumption of the differences) were employed to assess the final predictive efficacy.

## 3 RESULTS

### 3.1 Comparison of dose predictions for different networks

**FIGURE 4:**
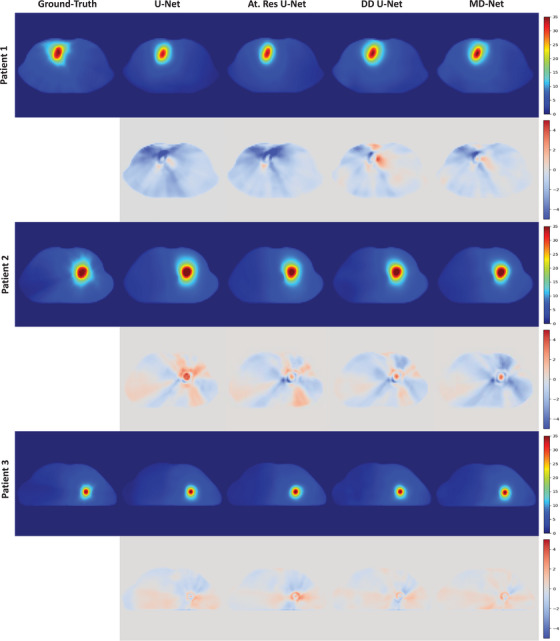
Dose prediction results using different networks (upper row) and the differences between the predicted results and the ground‐truth (lower row) for representative patients from the testing set. All dose distributions are illustrated in pseudocolor with a range of [0, 35] Gy, and all differences between the predicted results and the ground‐truth are illustrated in pseudocolor with a range of [‐5, 5] Gy.

In this section, we present the dose prediction results for different networks. Figure 4 illustrates the dose prediction outcomes of representative patients from the testing set using different 3D networks. The figure also shows the ground‐truth dose distributions along the disparities between the ground‐truth and predicted results for different networks. Table 2 lists the ADSs of the prediction results for different networks across different ROIs. The proposed MD‐Net achieved better ADSs in all ROIs except for the spinal cord, with an average value of 0.9595 across all ROIs. Table 3 lists the average absolute differences in the prediction results of the different networks for various dosimetric indices. MD‐Net exhibited excellent performance in predicting the dosimetric indices, particularly the GTV indices.

**TABLE 2:** Comparison of the average dose scores in different ROIs for different networks.

	$\mathrm{Average}\;\mathrm{dose}\;\mathrm{score}\;\left(ROI\right)=\frac{1}{k}\;\sum_{j=1}^{k}\frac{1}{n}\sum_{i=1}^{n}\left(\left|dose_{predict,\;ROI}^{i}-dose_{gt,ROI}^{i}\right|\right)\left(Gy\right)$			
ROI	U‐Net	At. Res U‐Net	DD U‐Net	MD‐Net
Heart	0.5013 ± 0.5107	0.4348 ± 0.3975	0.5044 ± 0.5054	**0.4254 ± 0.4361**
Lungs	0.6516 ± 0.3172	0.6377 ± 0.3278	0.6860 ± 0.3602	**0.5737 ± 0.2741**
Esophagus	0.6146 ± 0.3381	0.6148 ± 0.3788	0.6183 ± 0.3936	**0.5827 ± 0.4149**
Trachea	0.5787 ± 0.4550	0.6122 ± 0.4176	0.6502 ± 0.6154	**0.5761 ± 0.4109**
Spinal cord	0.5797 ± 0.3102	**0.5502 ± 0.2921**	0.6241 ± 0.3558	0.5725 ± 0.3133
PGTV	2.3435 ± 0.8910	2.3552 ± 0.7613	2.4892 ± 0.9915	**2.0629 ± 0.7002**
GTVb	2.4674 ± 0.6965	2.3941 ± 0.8349	2.2877 ± 1.0704	**1.9232 ± 0.5073**
Average of ROIs	1.1053 ± 0.9864	1.0856 ± 0.9729	1.1229 ± 1.0487	**0.9595 ± 0.7992**

All results are reported in the form of mean value ± standard deviation. Average dose scores shown in bold indicate the best results among the different networks.

**TABLE 3:** Comparison of the average absolute differences of the dosimetric indices for different networks.

		$\mathrm{Average}\;\mathrm{absolute}\;\mathrm{difference}\;\mathrm{of}\;\mathrm{dosimetric}\;\mathrm{indice}=\frac{1}{k}\sum_{j=1}^{k}\left(\left|DI_{predict}-DI_{gt}\right|\right)\left(Gy\right)$			
ROI	Dosimetric Indices	U‐Net	At. Res U‐Net	DD U‐Net	MD‐Net
Heart	*D*_mean_	0.364 ± 0.466	0.267 ± 0.299	0.295 ± 0.399	**0.257 ± 0.357**
Lungs	*D*_mean_	0.319 ± 0.278	**0.265 ± 0.210**	0.402 ± 0.372	0.269 ± 0.216
Esophagus	*D*_max_	1.403 ± 1.217	1.310 ± 1.221	1.017 ± 1.039	**1.010 ± 1.096**
	*D*_mean_	0.456 ± 0.327	**0.343 ± 0.273**	0.453 ± 0.331	0.372 ± 0.285
Trachea	*D*_max_	1.509 ± 1.712	1.542 ± 2.182	1.820 ± 2.484	**1.344 ± 1.249**
	*D*_mean_	0.415 ± 0.483	0.432 ± 0.425	0.520 ± 0.605	**0.409 ± 0.322**
Spinal cord	*D*_max_	1.420 ± 0.941	**1.245 ± 1.191**	1.411 ± 1.067	1.376 ± 0.887
	*D*_mean_	0.370 ± 0.322	0.401 ± 0.281	0.452 ± 0.357	**0.347 ± 0.343**
PGTV	*D*_max_	3.067 ± 0.831	2.517 ± 0.910	**2.137 ± 0.850**	2.495 ± 0.760
	*D*_mean_	1.296 ± 1.233	**1.196 ± 1.104**	1.682 ± 1.452	1.199 ± 0.804
	*D*_99_	1.244 ± 1.051	**1.123 ± 0.920**	1.349 ± 0.903	1.252 ± 1.035
	*D*_98_	1.179 ± 1.005	1.118 ± 0.838	1.373 ± 0.776	**1.117 ± 0.989**
	*D*_95_	1.312 ± 0.963	1.195 ± 0.931	1.434 ± 0.866	**1.091 ± 0.955**
GTVb	*D*_max_	3.067 ± 0.831	2.517 ± 0.910	**2.137 ± 0.850**	2.495 ± 0.760
	*D*_mean_	1.344 ± 0.937	1.263 ± 0.998	1.246 ± 1.245	**0.752 ± 0.618**
	*D*_99_	3.661 ± 3.151	4.023 ± 2.662	3.293 ± 2.885	**2.853 ± 1.642**
	*D*_98_	3.421 ± 2.479	3.373 ± 2.185	2.926 ± 2.383	**2.371 ± 1.421**
	*D*_95_	3.095 ± 1.962	3.200 ± 2.086	2.670 ± 1.889	**2.295 ± 1.082**

All results are reported in the form of mean value ± standard deviation. Average absolute differences of dosimetric indices shown in bold indicate the best results among the different networks.

### 3.2 Comparison of dose distributions for different loss functions

**FIGURE 5:**
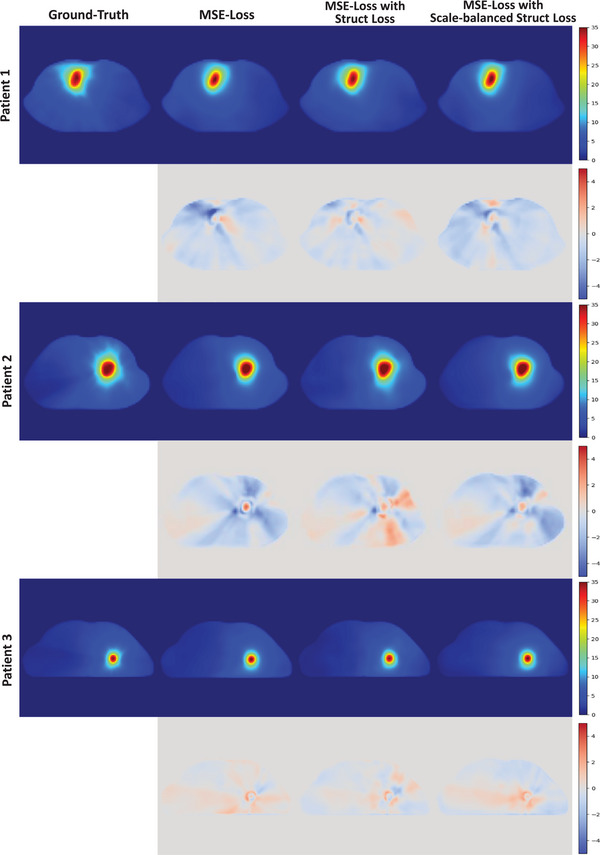
Dose prediction results using MD‐Net trained with different loss functions (upper row) and the differences between the predicted results and the ground‐truth (lower row) for representative patients from the testing set. All dose distributions are illustrated in pseudocolor with a range of [0, 35] Gy, and all differences between the predicted results and the ground‐truth are illustrated in pseudocolor with a range of [‐5, 5] Gy.

This section presents the prediction results of the MD‐Net trained with various loss functions. Figure 5 shows the dose prediction results for representative patients from the testing set using the MD‐Net trained with different loss functions. The ground‐truth dose distributions and differences between the ground‐truth and predicted results of the MD‐Net trained with different loss functions are also displayed. Table 4 lists the ADSs of the prediction results of the MD‐Net trained with different loss functions for various ROIs. The results indicate that the MSE‐Loss with a scale‐balanced structure loss achieved better ADSs (0.8837) compared with the results obtained using only MSE‐Loss (0.9595) or MSE‐Loss with the structure loss proposed by Koike et al.^27^ (0.9826). Table 5 lists the average absolute differences in the prediction results of the MD‐Net trained with different loss functions for various dosimetric indices. The results suggest that the MD‐Net trained with MSE‐Loss with a scale‐balanced structure loss achieved superior performance for most dosimetric indices.

**TABLE 4:** Comparison of the average dose scores for the MD‐Net trained with different loss functions.

	$\mathrm{Average}\;\mathrm{Dose}\;\mathrm{Score}\;\left(\mathrm{ROI}\right)=\frac{1}{k}\sum_{j=1}^{k}\frac{1}{n}\sum_{i=1}^{n}\left(\left|dose_{predict,\;ROI}^{i}-dose_{gt,ROI}^{i}\right|\right)\left(Gy\right)$		
ROI	MSE‐Loss	MSE‐Loss with structure loss	MSE‐Loss with scale‐balanced structure loss
Heart	**0.4254 ± 0.4361**	0.4641 ± 0.4358	0.4787 ± 0.5119
Lungs	**0.5737 ± 0.2741**	0.6258 ± 0.3469	0.6021 ± 0.2745
Esophagus	0.5827 ± 0.4149	0.6044 ± 0.5510	**0.5358 ± 0.4085**
Trachea	0.5761 ± 0.4109	0.5788 ± 0.5085	**0.4374 ± 0.3211**
Spinal cord	0.5725 ± 0.3133	**0.5674 ± 0.4289**	0.6001 ± 0.3716
PGTV	2.0629 ± 0.7002	2.1726 ± 0.9428	**1.9643 ± 0.6944**
GTVb	1.9232 ± 0.5073	1.8655 ± 0.6566	**1.5672 ± 0.6750**
ROIs average	0.9595 ± 0.7992	0.9826 ± 0.8821	**0.8837 ± 0.7523**

All results are reported in the form of mean value ± standard deviation. Average dose scores shown in bold indicate the best results among the MD‐Net trained with different loss functions.

**TABLE 5:** Comparison of the average absolute differences of dosimetric indices for different loss functions.

		$\mathrm{Average}\;\mathrm{absolute}\;\mathrm{difference}\;\mathrm{of}\;\mathrm{dosimetric}\;\mathrm{indice}=\frac{1}{k}\sum_{j=1}^{k}\left(\left|DI_{predict}-DI_{gt}\right|\right)\left(Gy\right)$		
ROI	Dosimetric indices	MSE‐Loss	MSE‐Loss with structure loss	MSE‐Loss with scale‐balanced structure loss
Heart	*D*_mean_	**0.257 ± 0.357**	0.305 ± 0.350	0.299 ± 0.469
Lungs	*D*_mean_	0.269 ± 0.216	0.341 ± 0.326	**0.237 ± 0.219**
Esophagus	*D*_max_	1.010 ± 1.096	1.197 ± 0.962	**0.586 ± 0.667**
	*D*_mean_	0.372 ± 0.285	0.451 ± 0.541	**0.351 ± 0.371**
Trachea	*D*_max_	1.344 ± 1.249	1.232 ± 1.689	**0.730 ± 0.735**
	*D*_mean_	0.409 ± 0.322	0.447 ± 0.537	**0.301 ± 0.341**
Spinal cord	*D*_max_	1.376 ± 0.887	**0.998 ± 0.862**	1.448 ± 0.818
	*D*_mean_	0.347 ± 0.343	**0.336 ± 0.420**	0.393 ± 0.316
PGTV	*D*_max_	2.495 ± 0.760	**1.135 ± 0.859**	1.395 ± 0.780
	*D*_mean_	1.199 ± 0.804	1.327 ± 1.184	**1.142 ± 0.816**
	*D*_99_	1.252 ± 1.035	1.097 ± 0.731	**1.001 ± 0.781**
	*D*_98_	1.117 ± 0.989	1.075 ± 0.738	**0.939 ± 0.787**
	*D*_95_	1.091 ± 0.955	1.159 ± 0.797	**0.950 ± 0.840**
GTVb	*D*_max_	2.495 ± 0.760	**1.135 ± 0.859**	1.395 ± 0.780
	*D*_mean_	**0.752 ± 0.618**	1.153 ± 0.752	0.870 ± 0.773
	*D*_99_	2.853 ± 1.642	2.381 ± 1.688	**2.256 ± 1.640**
	*D*_98_	2.371 ± 1.421	2.157 ± 1.398	**2.051 ± 1.353**
	*D*_95_	2.295 ± 1.082	2.107 ± 1.123	**1.837 ± 1.289**

All results are reported in the form of mean value ± standard deviation. Average absolute differences of the dosimetric indices shown in bold indicate the best results among the MD‐Net trained with different loss functions.

### 3.3 Comparison with the ground‐truth

**FIGURE 6:**
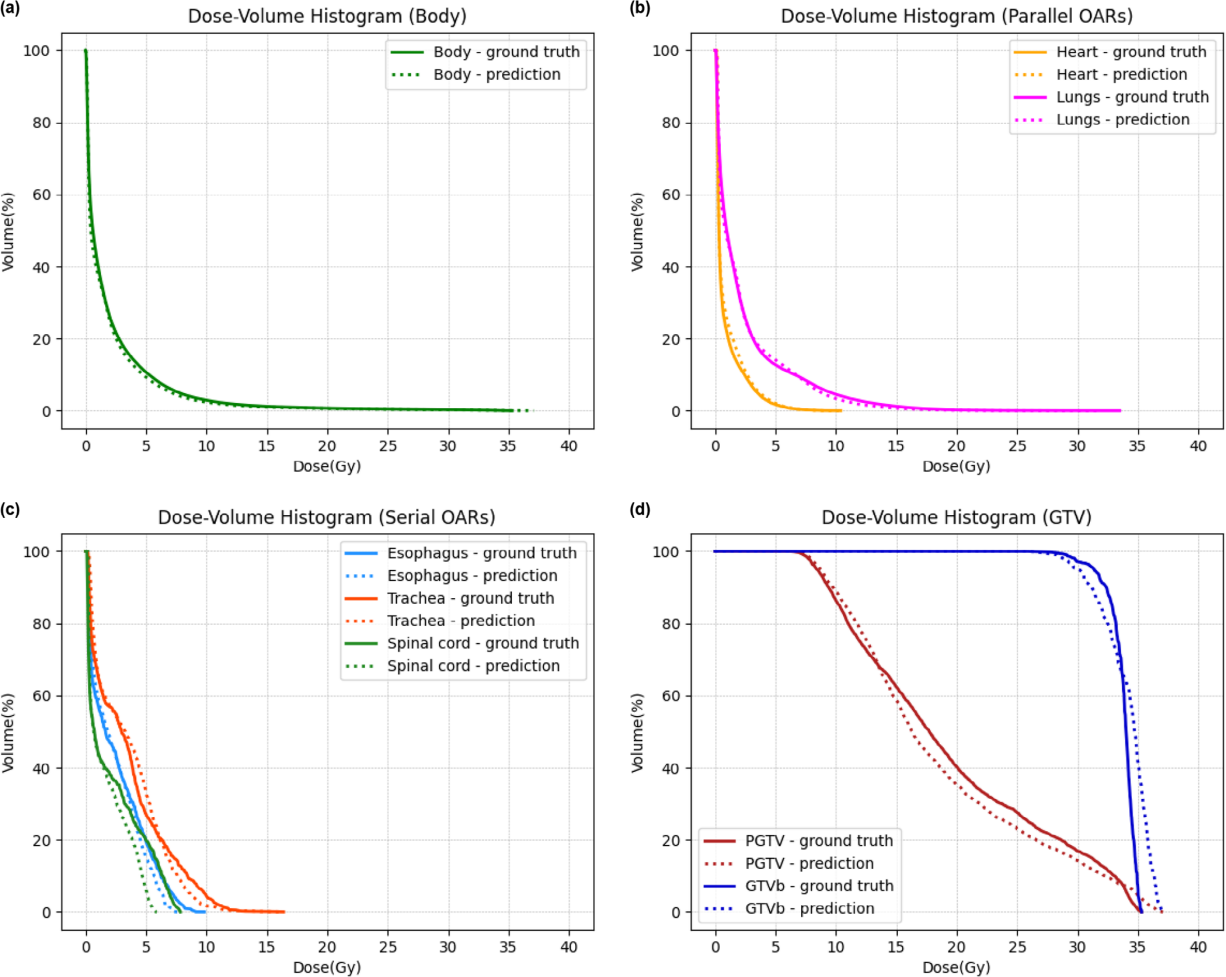
Dose‐volume histograms for a representative patient from the testing set. The four subgraphs show the ground‐truth (solid lines) and the results predicted by MD‐Net trained with MSE‐Loss with scale‐balanced structure loss (dashed lines) for the (a) body, (b) parallel OARs, (c) serial OARs, and (d) target volume.

To assess the effectiveness of the model, we present a comparative analysis of the prediction outcomes of our model (network: MD‐Net; loss function: MSE‐Loss with a scale‐balanced structure loss) and the ground‐truth. Figure 6 shows a comparison between the dose‐volume histogram (DVH) curves of different structures (including the body, parallel OARs, serial OARs, and target volume) for the ground‐truth and predicted plan of one representative patient from the testing set. Table 6 displays a detailed comparison between the ground‐truth and predicted plan across various dosimetric indices, along with the dose differences and corresponding *p*‐values. The results showed that, except for the $D_{mean}$ of the trachea, the $D_{max}$ of the spinal cord, and the target volume, there were no significant differences between the mean values of the other dosimetric indices for the ground‐truth and the predicted plan. The AADDIs (which reflect the predictive ability of the dosimetric indices) and ADSs (which reflect the accuracy of the dose prediction for each voxel) were elaborated in previous sections.

**TABLE 6:** Comparison of the dosimetric indices for the predicted and ground‐truth plans.

ROI	Dosimetric indices	Ground‐truth plans	Predicted plans	Dose differences	*p*
Heart	*D*_mean_	2.060 ± 1.955	1.800 ± 1.656	−0.261 ± 0.492	**0.117** ^b^
Lungs	*D*_mean_	2.909 ± 0.817	2.802 ± 0.957	−0.107 ± 0.305	**0.194** ^a^
Esophagus	*D*_max_	8.006 ± 3.271	7.806 ± 3.311	−0.200 ± 0.865	**0.900** ^b^
	*D*_mean_	2.943 ± 1.333	3.063 ± 1.547	0.120 ± 0.497	**0.365** ^a^
Trachea	*D*_max_	9.204 ± 5.878	9.607 ± 6.383	0.404 ± 0.954	**0.122** ^a^
	*D*_mean_	2.777 ± 1.711	2.994 ± 1.923	0.218 ± 0.400	0.044^b^
Spinal cord	*D*_max_	7.617 ± 2.265	6.720 ± 1.731	−0.897 ± 1.400	0.025^a^
	*D*_mean_	2.699 ± 1.207	2.522 ± 1.141	−0.177 ± 0.472	**0.166** ^a^
PGTV	*D*_max_	35.228 ± 0.778	36.091 ± 1.621	0.863 ± 1.346	0.025^a^
	*D*_mean_	19.110 ± 2.356	19.081 ± 1.729	−0.029 ± 1.403	**0.937** ^a^
	*D*_99_	7.796 ± 1.580	7.897 ± 1.235	0.101 ± 1.265	**0.762** ^a^
	*D*_98_	8.446 ± 1.657	8.548 ± 1.350	0.102 ± 1.221	**0.751** ^a^
	*D*_95_	9.419 ± 1.778	9.684 ± 1.534	0.265 ± 1.240	**0.420** ^a^
**GTVb**	*D*_max_	35.228 ± 0.778	36.091 ± 1.621	0.863 ± 1.346	0.025^a^
	*D*_mean_	32.797 ± 1.284	32.655 ± 1.572	−0.142 ± 1.155	**0.642** ^a^
	*D*_99_	25.502 ± 3.062	25.328 ± 2.419	−0.174 ± 2.784	**0.812** ^a^
	*D*_98_	26.957 ± 2.331	26.547 ± 2.362	−0.411 ± 2.422	**0.522** ^a^
	*D*_95_	29.003 ± 2.214	28.360 ± 2.076	−0.642 ± 2.150	**0.265** ^a^

^a^ The data meets the applicable conditions of the paired *t*‐test, and the paired *t*‐test is used for statistical analysis.

^b^ The Wilcoxon test is used for statistical analysis. *p*‐values shown in bold indicate no significant difference between the predicted and ground‐truth indices (*p* > 0.05).

## 4 DISCUSSION

In this study, we addressed the challenge of predicting the dose distributions in patients with bulky lung cancer undergoing P‐SABR treatment. We utilized MD‐Net, which is a 3D deep learning model tailored for this task. To accommodate diverse tumor sizes and distributions among patients, our model incorporates a multi‐scale input structure and multi‐scale feature extraction. By processing modules into the network, MD‐Net is capable of learning tumor features at different scales and perceiving different spatial ranges, thereby enhancing the dose distribution accuracy.

The standout characteristic of the network is that it maintains an encoder‐decoder structure similar to that of U‐Net while adding various modules that can capture and process multi‐scale information. This structure has a certain degree of targeting ability for tumor distribution characteristics and differences among patients with bulky lung cancers. In addition to our method, multiple other deep learning networks based on the U‐Net architecture have similar capabilities. For example, Attention U‐Net, proposed by Oktay et al.,^40^ can automatically learn features that focus on different shapes and scales, whereas Maji et al. further developed the field with their Attention Res U‐Net,^42^ which retains the advantages of the attention‐gating mechanism. Osman et al. applied the Attention Res U‐Net to 3D dose distribution prediction for head and neck radiotherapy, and the results indicated that this network significantly reduced the overall prediction error in GTVs and OARs compared with the Attention U‐Net.^18^ Gronberg et al. introduced the 3D Dense Dilated U‐Net, which employed consecutive dense connections with dilated convolutions to capture features at different scales.^19^ Compared with other advanced networks (DeepLabv3+, Hierarchically Densely Connected U‐Net, 3D V‐Net, and 3D U‐Net), 3D Dense Dilated U‐Net achieved superior performance in predicting the dose distributions in head and neck radiotherapy applications. We applied these two proven advanced networks to the dose distribution prediction of P‐SABR plans and compared them with our proposed MD‐Net. The comparison showed that MD‐Net exhibited higher accuracy on ROIs other than the spinal cord, with an ADS of 0.9595 for all ROIs, which was superior to those of the other networks (vs. 1.1053 for U‐Net, 1.0856 for At. Res U‐Net, and 1.1229 for DD U‐Net). The ADS in the spinal cord was only slightly inferior to that of the Attention Res U‐Net (0.5725 ± 0.3133 vs. 0.5502 ± 0.2921); however, it was still superior to other networks.

In the dose distributions of the P‐SABR plans for bulky lung cancer, we were also concerned with the dosimetric indices of OARs (i.e., heart, lungs, esophagus, trachea, and spinal cord) and GTVs (i.e., PGTV and GTVb) in the form of small absolute differences in predicting the major dosimetric indices, including the $D_{mean}$ of the heart; $D_{max}$ of the esophagus; $D_{max}$ and $D_{mean}$ of the trachea; $D_{mean}$ of the spinal cord; $D_{98}$ and $D_{95}$ of PGTV; and $D_{mean}$, $D_{99}$, $D_{98}$, and $D_{95}$ of GTVb. The transition from GTVb to PGTV entails rapid dose drops, posing challenges for accurate predictions. However, MD‐Net demonstrated superior performance in predicting the dose values of voxels within the GTV and the associated dosimetric indices.

In addition to optimizing the network structure, we examined the impact of loss functions on the dose distribution prediction. We introduced a structure loss that incorporates scale balance to enhance the performance of the dose distribution prediction methods. In previous dose distribution prediction methods, employing MSE‐Loss as the loss function for network training was a prevalent strategy.^11^, ^12^, ^13^ The MSE‐Loss provides equal attention to voxels within and outside ROIs, but clinical practice emphasizes voxel doses within ROIs. Previous studies have introduced the structural loss of the weighting of each ROI into the loss function to force the network to pay more attention to the voxels within the ROIs. Koike et al. introduced structural loss to predict the dose distribution of prostate cancer.^27^ The Dice score increased by more than 20% compared with that without structural loss, and the dose prediction errors in the PTV, rectum, and bladder were significantly reduced. Gronberg et al. used the MSE‐Loss with a weighted structure loss to predict the dose distribution in head and neck radiotherapy. The prediction results showed that the new loss function effectively improved the dose score compared with that achieved using only MSE‐Loss (from 2.77 to 2.65).^19^ This result indicates the effectiveness of introducing a structural loss to improve dose prediction performance. However, our bulky lung cancer patient dataset presents several challenges. First, fixed weights could bias results toward certain ROIs, neglect others, and affect the global dose distribution prediction. Second, adjusting fixed weights and managing high training costs are challenging when dealing with a large number of ROIs. Therefore, we propose a strategy with a scale‐balanced structure loss that has dynamic adaptability. Compared with fixed weights, this approach adaptively adjusts weights during training and better accommodates changes, which ensures that the ROI loss aligns with the global MSE‐loss, thereby preventing certain ROI losses from entering the overall loss and negatively impacting the overall dose distribution prediction. This introduces a mutual influence between the global MSE‐Loss and other structural losses, enhancing the generalizability of the model. We found several studies that incorporated dosimetric indices, or DVH, into the loss function to guide network training,^10^, ^26^ which helped improve the prediction accuracy of important indices and support clinical practice. In addition, the combination of clinical indicator loss and dose distribution loss helps alleviate model overfitting. However, compared with combining MSE‐Loss for all voxels and MSE‐Loss for each structure, dosimetric indices, DVH, and other new tasks included in the loss function have different data distributions and importance. Compared with simple weighting, which is difficult to design, using advanced multitask learning strategies, such as homoscedastic uncertainty,^29^ dynamic weight averaging,^30^ or gradient normalization,^31^ can be effective. This represents the focus of our future work.

Additionally, we compared the plans predicted using our most advanced method (MD‐Net trained with the MSE‐Loss with a scale‐balanced structure loss) with the ground‐truth P‐SABR plans. The results indicated that our proposed method accurately predicted the dose distributions, with statistically insignificant differences for most dosimetric indices. This suggests that MD‐Net can quickly generate accurate dose distributions and DVHs by processing the structural contours and CT images of patients with bulky lung cancer, potentially reducing the need for repeated dose calculations. Furthermore, the generated dose distributions and DVHs are directly applicable to guide radiation oncologists in adjusting the range of the GTVb, thus significantly enhancing the clinical efficiency of P‐SABR. However, comparison of the predicted DVHs for GTVb across patients revealed smoother curves, which posed challenges in determining the clinical dose for GTVb when compared to the ground‐truth. This issue will need to be addressed in future studies.

Despite these advancements, our study has certain limitations because of the difficulty in collecting P‐SABR plans for bulky lung cancers. Our data volume was relatively limited, although, based on advanced experience,^16^ we introduced data augmentation techniques (e.g., flipping, translation, and rotation) to compensate for the limited data. However, eliminating the negative impact of an insufficient sample size is difficult. In the future, additional data will be integrated to further improve the network prediction performance. Furthermore, because the delineation of GTVb does not solely rely on anatomical structure, the volume of GTVb may be further expanded with the advancement of precision radiotherapy technology. This could lead to a different data distribution for the new P‐SABR plans compared to the P‐SABR plans utilized in this study. Therefore, collecting new P‐SABR plans in the future will be necessary to mitigate the potential data bias and even improve the network based on the updated dataset. Moreover, the complex network structure of MD‐Net requires longer training times, increasing the training cost by approximately 25% compared with that of the classic U‐Net.

## 5 CONCLUSION

In this study, we proposed a novel dose prediction approach tailored to the unique data attributes of P‐SABR plans for bulky lung cancers. Enhancements include augmenting the 3D dose prediction network with multi‐scale feature extraction and processing capabilities, as well as refining the loss function to incorporate scale‐balanced structural losses. A comparative analysis of existing advanced dose prediction methods revealed notable enhancements in dose prediction accuracy, particularly for GTV. The experimental results underscore the potential of our proposed method for constructing precise dose prediction models, even with limited datasets. Moreover, the proposed model offers automated support for P‐SABR plans in cases of bulky lung cancer.

## AUTHOR CONTRIBUTIONS

Conception and design: Lei Huang, Xianshu Gao, Xuanfeng Ding. Acquisition of data: Lei Huang, Yue Li, Feng Lyu, Yan Gao, Yun Bai, Mingwei Ma, Siwei Liu, Jiayan Chen, Xueying Ren, Shiyu Shang. Analysis of data: Lei Huang. Writing the first draft of the manuscript: Lei Huang. All authors contributed to the drafting and editing of the manuscript and approved the final version.

## CONFLICT OF INTEREST STATEMENT

The authors declare no conflicts of interest.

## Data Availability

The data that support the findings of this study are available from the corresponding author upon reasonable request.
